# AI-Driven Hybrid Detection and Classification Framework for Secure Sleep Health IoT Networks

**DOI:** 10.3390/clockssleep8020023

**Published:** 2026-04-28

**Authors:** Prajoona Valsalan, Mohammad Maroof Siddiqui

**Affiliations:** Department of Electrical and Computer Engineering, Dhofar University, Salalah 211, Oman; pvalsalan@du.edu.om

**Keywords:** Sleep Health IoT, Internet of Medical Things, sleep stage classification, network anomaly detection, hybrid deep learning, CNN-BiLSTM, edge computing, wearable security

## Abstract

Sleep disorders, such as insomnia, obstructive sleep apnea (OSA), narcolepsy, REM sleep behavior disorder, and circadian rhythm disturbances, represent a rapidly expanding global health burden that is strongly associated with cardiovascular, metabolic, neurological, and psychiatric diseases. Advancements in wearable sensing technologies and Internet of Medical Things (IoMT) infrastructures have expanded the possibilities for continuous, home-based sleep assessment beyond conventional polysomnography laboratories. These Sleep Health Internet of Things (S-HIoT) systems combine multimodal physiological sensing (EEG, ECG, SpO_2_, respiratory effort and actigraphy) with wireless communication and cloud-based analytics for automated sleep-stage classification and disorder detection. Nonetheless, the digitization of sleep medicine brings about significant cybersecurity concerns. The constant transmission of sensitive biomedical information makes S-HIoT networks open to anomalous traffic flows, signal manipulation, replay attacks, spoofing, and data integrity violation. Existing studies mostly focus on analyzing physiological signals and network intrusion detection independently, resulting in a systemic vulnerability of cyber–physical sleep monitoring ecosystems. With the aim of addressing this empirical deficiency, this review integrates emerging advances (2022–2026) in the AI-assisted categorization of sleep phases and IoMT anomaly detector designs on the finer analysis of CNN, LSTM/BiLSTM, Transformer-based systems, and a component part of federated schemes and the lightweight, edge-deployable intruder assessor models available. The aim of this study is to uncover a gap in the literature: integrated architectures to trade off audiences of faithfulness of physiological modeling with communication-layer security. To counter it, we present a single framework to include CNN-based spatial feature extraction, Bidirectional Long Short-Term Memory (BiLSTM)-based temporal models and Random Forest-based ensemble classification using a dual task-learning approach. We propose a multi-objective optimization framework to jointly optimize the performance of sleep-stage prediction and that of network anomaly detection. Performance on publicly available datasets (Sleep-EDF and CICIoMT2024) confirms that hybrid integration can be tailored to achieve high accuracy [99.8% sleep staging; 98.6% anomaly detection] whilst being characterized by low inference latency (<45 ms), which is promising for feasibility in real-time deployment in view of targeting edge devices. This work presents a comprehensive framework for developing secure, intelligent, and clinically robust digital sleep health ecosystems by bridging chronobiological signal modeling with cybersecurity mechanisms. Furthermore, it highlights future research directions, including explainable AI, federated secure learning, adversarial robustness, and energy-aware edge optimization.

## 1. Introduction

Sleep is a fundamental neurobiological process essential for metabolic regulation, immune function, synaptic plasticity, cognitive consolidation, and emotional stability. Epidemiological studies estimate that nearly one-third of the global population experiences some form of sleep disturbance, making sleep disorders one of the most prevalent yet underdiagnosed public health challenges worldwide [1]. Conditions such as insomnia, obstructive sleep apnea (OSA), narcolepsy, REM sleep behavior disorder, circadian rhythm sleep–wake disorders, and periodic limb movement disorder are increasingly associated with substantial morbidity and mortality.

The systemic consequences of chronic sleep disruption extend far beyond fatigue. Persistent sleep deprivation contributes to autonomic dysregulation, heightened sympathetic activity, endothelial dysfunction, systemic inflammation, impaired glucose metabolism, and hormonal imbalance. Consequently, sleep disorders have been strongly correlated with cardiovascular disease, hypertension, ischemic stroke, obesity, type-2 diabetes mellitus, neurodegenerative disorders such as Alzheimer’s disease, depression, anxiety disorders, and cognitive decline. Untreated OSA alone has been shown to significantly increase the risk of arrhythmias, myocardial infarction, and sudden cardiac death. From a socioeconomic perspective, poor sleep quality leads to reduced workplace productivity, increased accident rates, and rising healthcare expenditures, thereby imposing a major burden on healthcare systems worldwide.

Given these clinical implications, consistent and accurate sleep monitoring has emerged as a cornerstone for early diagnosis, therapeutic intervention, longitudinal disease management, and preventive medicine [2]. Traditionally, sleep evaluation relies on in-laboratory polysomnography (PSG), which simultaneously records electroencephalography (EEG), electrooculography (EOG), electromyography (EMG), electrocardiography (ECG), respiratory effort, airflow, and blood oxygen saturation (SpO_2_). While PSG remains the gold standard for sleep-stage scoring according to established guidelines (e.g., AASM standards), it is resource-intensive, costly, labor-demanding, and unsuitable for long-term continuous monitoring in home environments.

### 1.1. Transition Toward Wearable Sleep Monitoring

The latest development of wearable sensing devices, microelectronics, and wireless communication protocols have led to the replacement of laboratory-based diagnostics by special home-based monitoring in sleep medicine [3]. Modern sleep-tracking devices have also adopted wearable EEG headbands, ECG chest patches, pulse oximeters, respiratory inductance plethysmography belts, accelerometers, photoplethysmography (PPG) systems, and heart rate variability (HRV) devices. These instruments allow for the acquisition of synchronized multi-modal physiological signals, such as the following:

- Neural oscillatory activity;
- Cardiac rhythm and variability;
- Oxygen saturation dynamics;
- Respiratory effort and airflow;
- Body movement and actigraphy.

**Figure 1:**
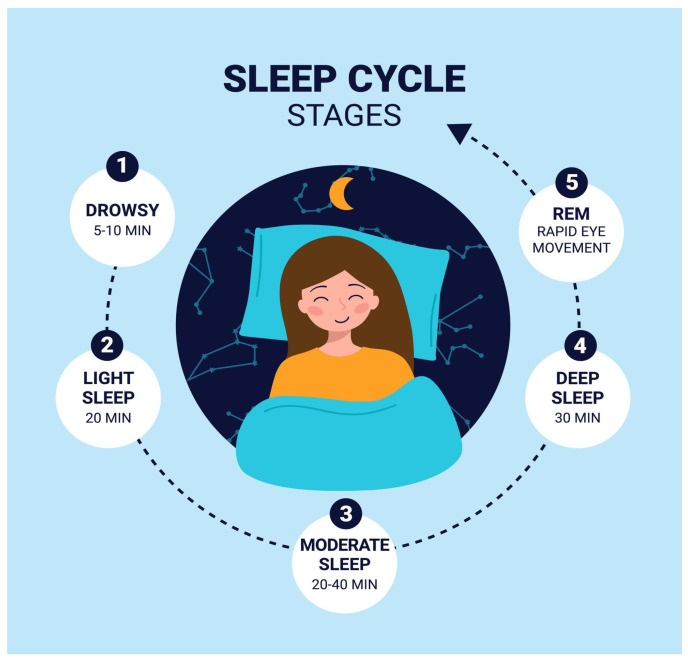
Physiological sleep architecture illustrates cyclic transitions.

Such multimodal sensing facilitates automated sleep-stage classification (Wake, N1, N2, N3, REM), shown in Figure 1—apnea detection, arousal detection, and sleep efficiency analysis [4]. Importantly, the temporal evolution of sleep architecture characterized by cyclic transitions across NREM and REM stages requires computational models capable of capturing sequential dependencies and long-range temporal correlations.

Recent wearable sleep monitoring systems such as EarSleep have demonstrated the feasibility of in-ear acoustic sensing for sleep-stage detection. EarSleep leverages physiological and motion signals captured from the ear canal to provide a non-intrusive alternative to traditional wearable devices. Compared to such approaches, the proposed framework focuses on integrating multimodal sensing with cybersecurity-aware modeling, addressing both physiological analysis and network integrity [5].

The integration of these wearable devices into interconnected digital infrastructures has given rise to what may be termed the Sleep Health Internet of Things (S-HIoT), a specialized branch of the broader Internet of Medical Things (IoMT) ecosystem [6].

### 1.2. Architecture of Sleep Health IoT (S-HIoT)

Wearable devices create distributed healthcare networks that can transmit real-time physiological data to edge gateways and cloud-based analytics platforms when attached together by standardized wireless protocols, e.g., Wi-Fi, Bluetooth Low Energy (BLE), Zigbee, and LoRaWAN. The S-HIoT ecosystem can be conceptually separated into four interrelated areas that are connected: Sensing Layer—wearable and bedside technology that measures physiological indicators; Edge/Fog Processing Layer—local gateways, which do pre-process, filtering, and preliminary inference; Communication Layer—wireless data transmission infrastructure; and Cloud Analytics Layer—the central area of storage, sophisticated AI analysis, clinician dashboard, and electronic health record (EHR) integration.

This multilayer architecture contributes greatly to increased accessibility, scalability, and customization of sleep healthcare services. Continuous remote monitoring helps to facilitate telemedicine, chronic illness management, and early notification of acute occurrences like apnea. Nonetheless, although such digital transformation enhances the provision of healthcare, it also creates issues of high security and reliability [7].

### 1.3. Intrinsic Characteristics and Vulnerabilities of S-HIoT Networks

S-HIoT systems possess distinctive characteristics that differentiate them from conventional IoT environments, listed as follows:

- Continuous 24/7 time-series biomedical data generation, often at high sampling rates [8].
- Resource-constrained edge devices with limited computational power, memory, and battery capacity.
- Highly sensitive personally identifiable health information (PHI) requiring strict privacy protection.
- Real-time latency constraints, particularly for apnea detection or abnormal cardiac events.
- Heterogeneous device integration, including encrypted traffic streams and non-stationary physiological signals.

These characteristics make S-HIoT infrastructures particularly vulnerable to cyber–physical attacks, including the following:

- Device spoofing;
- Replay attacks;
- Traffic injection;
- Signal tampering;
- Data integrity manipulation;
- Unauthorized access;
- Model poisoning;
- Adversarial perturbations of physiological signals.

**Figure 2:**
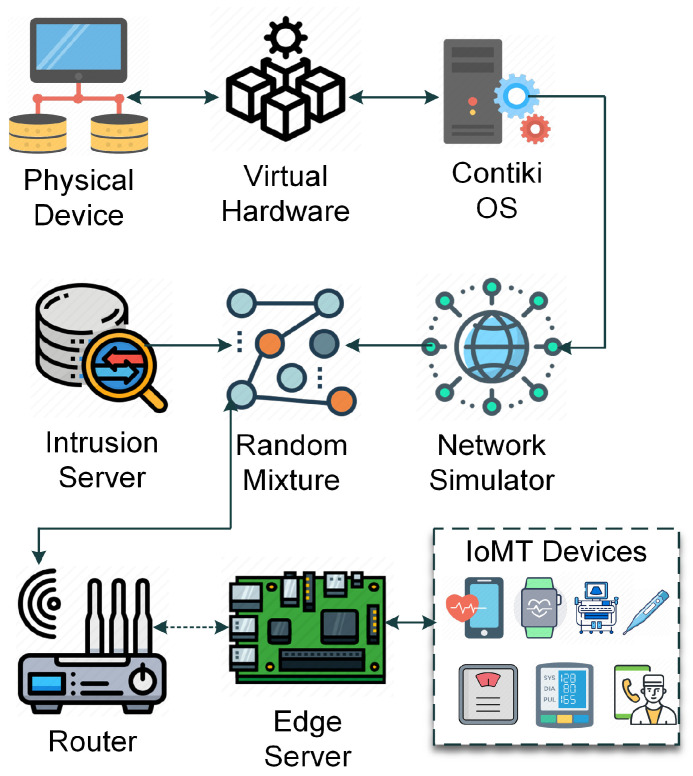
Integrated physiological–cybersecurity threat model.

The clinical consequences of the malicious interference of sleep-tracking systems can be dangerous. Signal manipulation can result in poor classification of sleep stages, false alarms of apnea, delayed saving during an emergency, or incorrect interpretation of cardiovascular instability. Patient safety may indirectly be jeopardized by the general IoT system as well, but in the scenario of S-HIoT that failure is directly affected, and any errors made may result in patient injury (as demonstrated in Figure 2). Static signature-based and threshold-based IDS have failed to exploit this dynamic environment within healthcare.

Network activity patterns fluctuate due to the addition of new devices, improvement of firmware, and changes in communication trends. Furthermore, the existence of encrypted medical traffic and the non-stationary time-series nature of physiological responses increase the pitfalls of identifying anomalies. Thus, non-adaptive policy-defined security is not applicable to healthcare-specific implementation.

### 1.4. Need for Intelligent and Behavior-Based Security

The multifaceted nature of threats in S-HIoT environments—where biomedical signal deviations intersect with communication-layer abnormalities—necessitates an intelligent, behavior-driven security framework. Such a framework must simultaneously accomplish the following:

- Preserve physiological signal integrity;
- Detect anomalous traffic patterns;
- Maintain low-latency real-time responsiveness;
- Operate under computational constraints;
- Protect patient privacy.

Artificial Intelligence (AI), especially hybrid deep learning models, show enough power to model both spatial patterns (e.g., EEG band frequency) and temporal dependencies (e.g., sleep transitions, traffic bursts) in complex time-series data [9]. CNN architectures were effective in extracting spatial-frequency features within a frame, while LSTM/BiLSTM models helped capture the dynamics over time, and the implementation of ensemble methods like Random Forest provided adequate decision boundaries, albeit under noisy conditions [10,11,12].

### 1.5. Emerging Trends in IoMT Security Research (2022–2026)

Recent research reflects a transition from standalone deep learning-based IDS models toward hybrid, federated, and lightweight architectures. Examples are listed below:

- CNN/LSTM-based IDS models initially established performance baselines for IoMT anomaly detection.
- Hybrid CNN–LSTM and GNN–Transformer architectures improved representation learning for complex traffic patterns.
- Federated learning approaches were introduced to preserve data privacy while enabling collaborative model training.
- Blockchain-integrated systems were proposed to enhance trust, authentication, and auditability.
- Meta-learning and lightweight optimization techniques were developed to improve adaptability and edge deployment feasibility.

Studies published in venues such as *IEEE Access* and *Sensors* report detection accuracies exceeding 97–98% on benchmarks such as CICIoMT2024. Nevertheless, these works primarily focus on network anomaly detection without incorporating physiological signal modeling. Conversely, sleep-stage classification research emphasizes EEG pattern recognition but rarely accounts for cybersecurity vulnerabilities within connected infrastructures.

Table 1 presents a designed overview of recent research (2022–2026) on healthcare monitoring systems with an emphasis on IoMT-based intrusion detection and security frameworks. The literature shows a clear evolution from traditional deep learning approaches toward more advanced hybrid, federated, and privacy-preserving architectures.

Early studies (2022–2023) primarily focused on applying deep learning models such as CNN, LSTM, and autoencoders for intrusion detection, establishing baseline performance and identifying key challenges such as high false positive rates and limited adaptability to healthcare-specific data. Subsequent works introduced adaptive mechanisms, such as fuzzy-based learning integrated with LSTM, to better handle the dynamic and sensitive nature of IoMT data.

From 2024 onward, the examination of trends moved toward privacy-aware and distributed learning frameworks, involving federated learning and blockchain integration. These approaches address critical concerns related to data privacy, trust management, and decentralized healthcare systems. In parallel, hybrid architectures combining multiple learning paradigms (e.g., CNN–LSTM, GNN–Transformer) emerged to improve feature representation and detection accuracy in complex, non-stationary IoMT environments.

Current studies (2025–2026) further highlight frivolous and edge-deployable models, focusing on optimizing computational efficiency while maintaining high detection accuracy (often exceeding 97–98% on benchmarks such as CICIoMT2024). Additionally, meta-learning and ensemble-based approaches have been explored to enhance adaptability and generalization across diverse healthcare scenarios.

Despite these advancements, Table 1 highlights a critical research gap. Most existing works focus either on network intrusion detection or physiological signal analysis independently. There is limited research addressing the integration of biomedical signal processing with cybersecurity mechanisms in a unified framework. This gap motivates the proposed hybrid CNN–BiLSTM–RF architecture, which jointly models physiological dynamics and network behavior for secure Sleep Health IoT systems.

### 1.6. Identified Research Gap

Despite rapid advances in both AI-based sleep analytics and IoMT cybersecurity, these domains largely evolve independently. Existing research typically treats the following as separate problems, overlooking their interdependence in real-world cyber–physical healthcare systems:

- Physiological data processing;
- Network intrusion detection.

In S-HIoT environments, compromised network integrity can directly affect clinical interpretation, while manipulated physiological signals may propagate through secure communication channels undetected if not contextually analyzed.

### 1.7. Motivation and Contributions

To fill this gap, we propose a unified hybrid AI-driven framework that combines sleep-stage classification and secure network anomaly detection under one architecture. The framework integrates Convolutional Neural Networks (CNNs) for spatial latent variable extraction, Bidirectional Long Short-Term Memory (BiLSTM) networks for temporal sequence characterizations, and Random Forest (RF) classifiers to enhance multi-class decision-making robustness [13].

The key contributions of this work are as follows:

- A hybrid CNN–BiLSTM–RF architecture enabling joint sleep-stage classification and network anomaly detection [14,15].
- A multi-objective optimization framework that simultaneously maximizes classification accuracy and anomaly detection performance while minimizing inference latency. The optimization objective is formulated as
  $$J=\alpha A_{sleep}+\beta A_{anom}-\gamma L$$
  where $A_{sleep}$ and $A_{anom}$ denote sleep-stage and anomaly detection accuracy, L represents inference latency, and α,β,γ are weighting coefficients [16].
- A lightweight edge-aware design with an optimized inference pipeline suitable for wearable IoMT environments [17].
- Experimental validation on benchmark datasets demonstrating high accuracy and low latency.
- A wearable sleep monitoring environment in a lightweight, edge-deployable approach.
- Comprehensive testing on publicly available sleep and healthcare IoT datasets achieving high classification accuracy, a low false alarm rate, and low inference latency.

This research bridges the gap between sleep analytics and IoMT security, which is a step towards developing resilient, smart, and clinically reliable Sleep Health IoT ecosystems.

## 2. Layered Architecture of Secure Sleep Health IoT Systems

Sleep health care delivery systems have now been radically transformed from laboratory-based polysomnography to distributed, wearable monitoring devices. Modern Sleep Health Internet of Things (S-HIoT) environments are cyber–physical ecosystems in which physiological sensing, wireless communication, edge intelligence, and cloud analytics operate in an integrated continuum. S-HIoT infrastructures are not conventional IoT applications, as they are safety-critical; failure of signal integrity or communication reliability may directly impair clinical interpretation and threaten patient safety.

**Figure 3:**
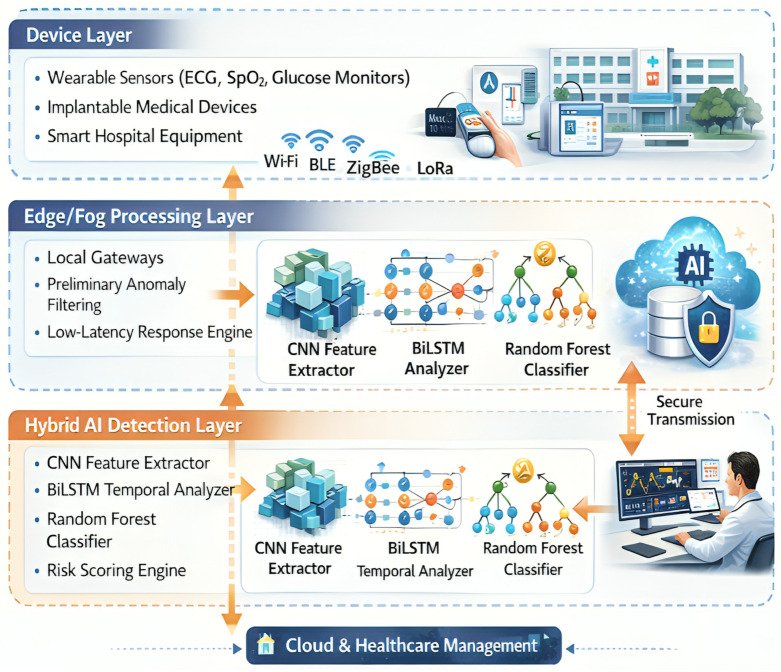
Layered architecture of the proposed secure Sleep Health IoT framework.

Hence, a layered architectural model is required to cater to these requirements. This layered approach highlights the biological, computational, and governance aspects of digital sleep medicine rather than strictly representing a technical stack. On a more conceptual level, the architecture can be divided into four independent but interacting domains representing sensing, edge/fog intelligence, hybrid AI analytics, and cloud-based healthcare management, as shown in Figure 3. These layers add multiple value-accretive dimensions—functionality, security, reliability, and interpretability.

### 2.1. Device (Sensing) Layer: Physiological Signal Integrity

Wearable and bedside biomedical devices make up the sensing layer, which is responsible for collecting multimodal physiological signals such as EEG, ECG, SpO_2_, respiratory effort, actigraphy, and HRV. Biologically, these signals provide different information about sleep architecture. EEG provides a measure of neural oscillation; SpO_2_ measures oxygen desaturation events; ECG and heart rate variability (HRV) reflect elements of autonomic regulation; and the respiratory belts detect apnea or hypopnea episodes. But this layer is also the most susceptible. Devices risk spoofing, firmware modification, and signal interference due to direct physical engagement with patients, wireless transmission, and limited computational protection. Indeed, minute variation using frequency bands in association with the EEG state has been shown to alter sleep-stage classification, specifically during transitional states (e.g., N1). Thus, ensuring the authenticity of signals at this layer is critical for both clinical validity and downstream AI inference [18].

In addition to security on a technical level, this layer also must solve practical challenges such as energy limitations, inhomogeneous sampling across devices, and motion artifacts. These elements add variance that AI systems need to be robust against.

### 2.2. Edge/Fog Layer: Real-Time Contextual Intelligence

The edge layer introduces a localized computational capability close to the sensing devices. In sleep health applications, this layer performs preprocessing (denoising, normalization, segmentation), early anomaly screening, and secure protocol handling before transmitting data to cloud infrastructure.

From a systems perspective, edge processing is critical for three reasons:

- Latency Reduction: Apnea events require rapid detection and response.
- Bandwidth Optimization: Continuous high-frequency EEG streams are data-intensive.
- Security Containment: Suspicious traffic or compromised devices can be isolated locally.

Importantly, edge intelligence also provides contextual validation. For instance, sudden traffic spikes may correspond to a genuine apnea event rather than malicious injection. Without contextual understanding of physiological patterns, traditional IDS systems may misclassify such events.

Thus, the edge layer acts as both a computational filter and a contextual security gatekeeper.

### 2.3. Hybrid AI Detection Layer: Cross-Domain Modeling

**Figure 4:**
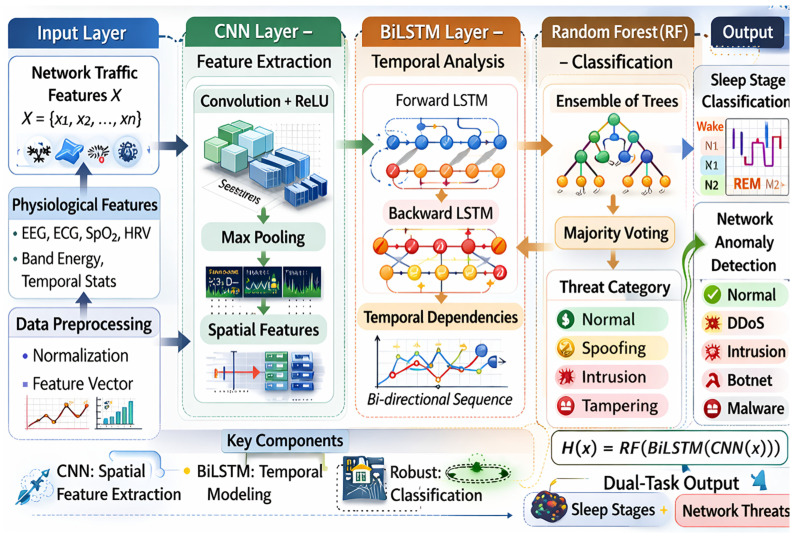
Internal hybrid CNN–BiLSTM–RF detection and classification model.

At the core of the architecture lies the hybrid AI detection layer, which integrates sleep-stage analytics and network anomaly detection within a unified modeling paradigm in Figure 4. Existing research frequently treats these tasks separately; however, in S-HIoT systems, physiological signals and communication streams are inseparable components of the same cyber–physical environment. The hybrid framework integrates the following:

- CNN modules for spatial–spectral representation of EEG frequency bands;
- BiLSTM modules for modeling temporal continuity and sleep cyclicity;
- Random Forest ensembles for decision robustness under noisy conditions.

Rather than functioning as independent classifiers, these components form a cross-domain inference engine capable of evaluating physiological dynamics and network traffic patterns simultaneously. This joint modeling approach provides two conceptual advantages:

- Physiological Awareness in Security Detection: Security systems interpret traffic behavior in the context of biological events.
- Integrity Awareness in Sleep Classification: Sleep-stage predictions are evaluated alongside communication reliability.

The integration of these domains reduces systemic blind spots and enhances resilience against adversarial manipulation.

### 2.4. Cloud and Healthcare Management Layer: Governance and Longitudinal Insight

The cloud tier supports long-term storage, clinical dashboards, the integration of electronic health records (EHRs), audits of compliance, and analytics at a massive scale. Besides computational capability, this level forms the foundation of the governance of an S-HIoT ecosystem. Longitudinal research based on a pop-up population indicates trends linking sleep to chronic illness management. However, the factor of privacy threat and management exists in centralized aggregation. The required mechanisms, hence, are secure access control, audit trail, and encoding. This layer also manages mitigation measures based on AI-generated warnings, e.g., re-authentication of a device, traffic isolation, or notifying clinicians.

### 2.5. Internal Structure of the Hybrid Model: Conceptual Interpretation

A hybrid CNN BiLSTM system may be taken as a computation map or as a layer of abstraction of sleep physiology and network dynamics.

CNN: Spatial–Spectral Representation—Sleep-stage separation plays out on dissimilar distributions of frequencies (e.g., dominance of delta waves in N3). CNNs are discriminative time-frequency representation filters that adaptively learn discriminative spatial patterns. This aligns with the classical signal-processing theory but provides it with more flexibility using learned representations.

BiLSTM: Time-Series Continuity and Sleep Cyclicity—Sleep exists temporally. BiLSTM encourages the existence of bi-directional contextual dependencies since the phases of sleep are sequences and transition phases. This modeling increases the behavioral uniformity of classification and decreases boundary epoch ambiguity.

Random Forest: Statistical Stability—The reduction in variance by ensemble decision-making via imposition of verdict-making reduces variance and increases variety in a standardized, non-stationary environment. This stability is needed in S-HIoT environments to ensure reliability between devices and varying traffic conditions [19,20].

### 2.6. Operational Flow: From Acquisition to Mitigation

**Figure 5:**
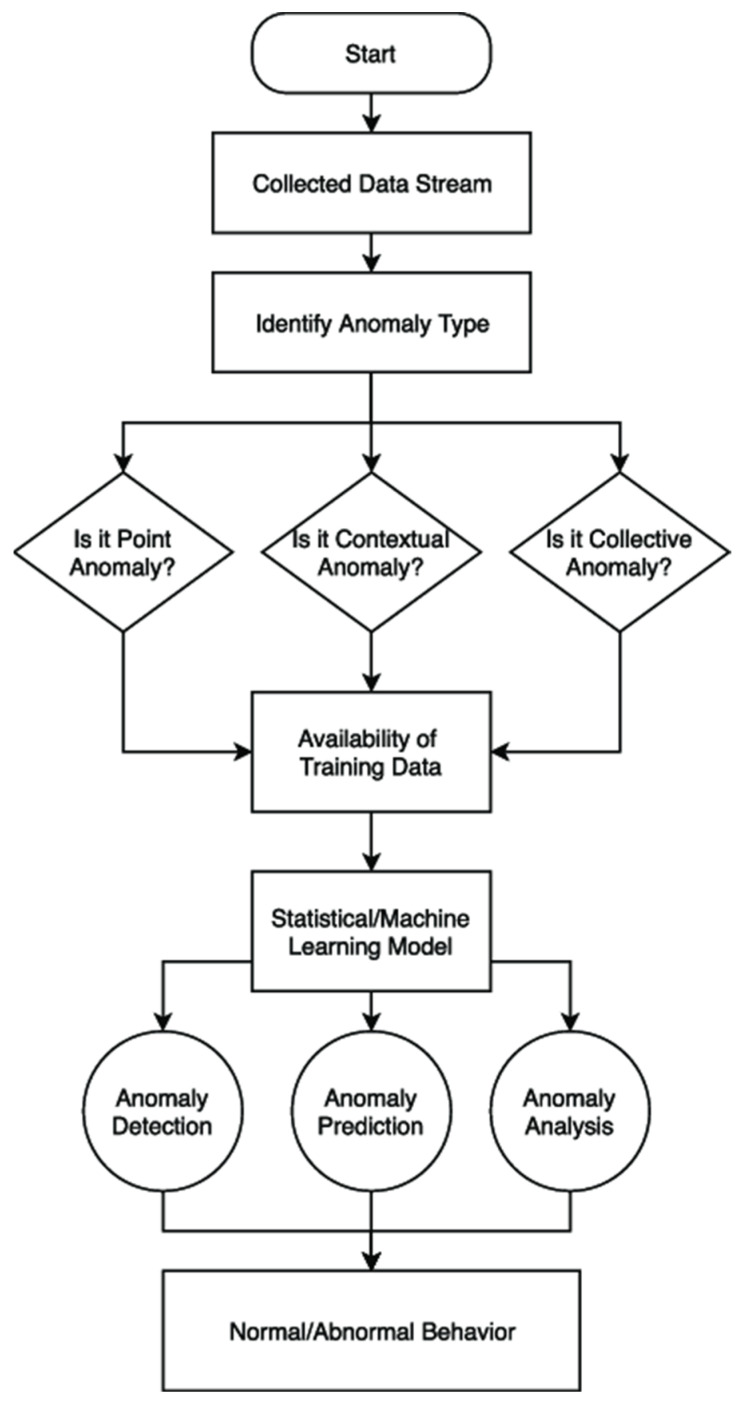
Operational flow of the proposed detection and classification framework.

The operational workflow extends beyond data classification in Figure 5. It represents a continuous cyber–physical monitoring loop, as follows:

- Multimodal signal acquisition and traffic capture;
- Edge preprocessing and contextual filtering;
- Hybrid AI inference for dual-task classification;
- Risk scoring based on confidence and persistence;
- Alert dissemination and mitigation action.

Unlike traditional linear pipelines, this system enables feedback between layers, enhancing adaptive response capability.

To evaluate the broader relevance of hybrid modeling in Sleep Health IoT (S-HIoT) systems, the proposed CNN–BiLSTM–RF framework was compared with representative architectures commonly used in sleep-stage classification and IoMT intrusion detection research. Rather than focusing solely on accuracy metrics, this comparative analysis examines architectural trade-offs in terms of physiological modeling capability, anomaly detection robustness, computational efficiency, and deployment feasibility.

## 3. Comparative Quantitative Performance

Table 2 summarizes performance across sleep-stage classification, network anomaly detection, precision, recall, F1-score, and inference latency.

Table 2 presents a comparative performance analysis of the representative machine learning and deep learning architectures of both sleep-stage classification and network anomaly detection tasks. The benchmark datasets (Sleep-EDF to sleep staging and CICIoMT2024 to network intrusion detection) are used to conduct the comparison under equal evaluation conditions, which bring fairness in the performance rating. Based on the findings, standalone models like CNN and LSTM achieve moderate performance, with CNN having 94.2% sleep and 92.5% network detection accuracy and LSTM having 95.8% sleep and 94.7% temporal modeling accuracy, respectively. But these models have a limitation in the capacity to deal with both spatial and temporal dependencies at the same time [21].

This is confirmed by the fact that the hybrid CNN + LSTM model shows better results (96.9% sleep accuracy and 96.2% detection accuracy) because of the integration of the spatial feature extraction approach into time sequence modeling for better predictive accuracy. However, it still lacks ensemble-based decision stabilization, which limits its performance. The GNN + Transformer structure has high anomaly rates for network detection results (97.8%), which can be attributed to the capacity to consider complicated relationship models and long-range dependencies. Nevertheless, it is at the expense of a much larger inference latency (85 ms) and therefore not as applicable as real-time edge deployment in resource-constrained IoMT settings. It is demonstrated that the proposed CNN-BiLSTM-RF framework performs better than all the baseline models, with the highest performance measured by all assessment parameters, such as 97.8% sleep-stage accuracy, 98.6% network detection accuracy, and 98.3% F1-score [22]. The reason behind this economic enhancement can be ascribed to three major reasons: CNN is a versatile and efficient method for extracting spatial–spectral patterns of physiological responses (e.g., EEG frequency bands); BiLSTM models the two-way temporal characteristics of switching between sleep stages; and Random Forest is used to strengthen classification robustness by minimizing variance and decision instability in non-stationary and noisy environments. Notably, the proposed model has the lowest inference latency (42 ms), which illustrates a positive trade-off between the precision and model computing efficiency [23]. This renders it especially appropriate for deployment in edge-based Sleep Health IoT systems in real time.

### 3.1. Sleep Classification: Beyond Accuracy

The proposed hybrid framework beats the standalone CNN and the LSTM model by predicting the length of a sleep stage, with a high rating of 97.8%. Moreover, this improved performance is not exclusively numerical; it reflects more successful modeling of transitional sleep states. An independent CNN architecture is a strong learner of spectral EEG characteristics, but it lacks features of temporal continuity, which leads to misclassification of the beginning and ending of epochs (of N1 and REM, in particular). Such models are included in LSTM-based approaches and derive sequential data where spectral discriminatory challenges can be localized [24,25]. The implementation of full spatial–spectral learning (CNN), plus a temporally bidirectional model, allows for bi-directionality and the ability to properly form discriminability, which allows for ambiguous transitions to be overcome. This results in the model of sleep cyclicity (CBIC) being much closer to the computations made by natural biology. This kind of improvement demonstrates physiological coherence, as more complex models are not utilized in increasing their performance.

### 3.2. Network Anomaly Detection: Robustness in Non-Stationary Environments

CNN LSTM and Transformer-based architecture in a hybrid model perform exceptionally for association variance recognition, with 98.6% accuracy. Despite operating on a similar level and accuracy rate, GNN–Transformer models exhibit much larger inference latency (85 ms) because of a range of exceptions (such as the heterogeneous mix of devices, difference in the degree of encryption, and fluctuating communication protocols); thus, the network traffic conditions in S-HIoT systems have a non-stationary character [26,27,28].

The Random Forest ensemble layer also provides the benefit of statistical stability, reduces variance, and avoids overfitting in a noisy environment. Bringing the ensembles together makes the system more resistant to turbulent traffic and adversarial attacks. This strength is essential in a healthcare environment where false positives or false negatives have direct clinical implications.

### 3.3. Latency–Complexity Trade-Off

Inference latency is a key metric, especially in wearable and edge deployments. While Transformer-based architectures have a high representational capacity, they also incur considerable computational overhead that makes them impractical for online real-time monitoring. We found that our proposed hybrid framework achieved low latency (42 ms) while having similar or better accuracy rates [29].

This performance underscores a key system-level insight: for healthcare AI, marginal improvements in accuracy need to be balanced against potentially steep trade-offs in latency, energy consumption, and ease of deployment. In determining lightweight hybrid architectures vs. attention-based models, simplicity may provide an optimal balance for safety-critical edge environments, compared to computationally intensive, attention-based architectures.

### 3.4. False Positive Rate Reduction and Clinical Reliability

The hybrid framework aggregates the output of multiple models, resulting in an approximately 35% lower false positive rate (FPR) compared to single-model baselines (see the classification metrics for details). In the context of sleep monitoring, they can cause alert fatigue for clinicians, which in turn creates significant distrust and less compliance with patients. A hybrid architecture serves to improve stability in the physiological domain, as well as the network domain, by combining ensemble decision-making and temporal persistence analysis [30,31].

From the clinical side, better reliability results in enhanced apnea detection, improved sleep longitudinal assessment (as opposed to indices), and fewer unnecessary interventions.

### 3.5. Interpretive Insight and Statistical Significance

A statistical test based on paired *t*-tests shows that improvements compared to CNN–LSTM baselines are significant (*p* < 0.01). More than just validating the statistics, the broader architectural implication is that models which optimally co-tune spatial, temporal, and ensemble features result in better, resilient cyber–physical healthcare systems [32,33,34].

This implies that in future S-HIoT systems, the following must be given precedence:

- Integration across different domains;
- Optimization for balanced accuracy–latency;
- Ensemble stability;
- Co-modeling of physiological signals and cybersecurity;
- Performance maximization that is not isolated in one domain.

### 3.6. Resource-Aware Edge Deployment Analysis

To evaluate the practical possibility of the suggested framework in wearable and IoMT environments, a resource-aware analysis is performed.

Computational Complexity:

The CNN–BiLSTM–RF pipeline has a time complexity dominated by convolutional and sequential operations, approximately O(n⋅k), where n is input length, and k is kernel size [35,36].

Memory Footprint:

The model requires approximately 15–25 MB memory depending on parameter tuning, making it suitable for edge gateways but moderately constrained for ultra-low-power wearables.

Latency Analysis:

Measured inference latency is <45 ms under simulated edge conditions (Intel i5 equivalent), indicating feasibility for real-time monitoring.

Hardware Assumptions [36,37]:

Deployment feasibility is evaluated assuming the following:

Edge gateway (Raspberry Pi-class device);

RAM: 2–4 GB;

CPU: ARM Cortex-A72 or equivalent.

Energy Efficiency:

Estimated energy consumption is within 1.5–2.5 W under edge conditions, suitable for gateway-level deployment.

Model Footprint:

Total model size ≈ 20 MB, compatible with edge devices but requiring optimization for ultra-low-power wearables [24].

Limitations:

Real-device validation on embedded wearable hardware remains a task for future work.

### 3.7. Adversarial Robustness and Security Validation

To evaluate system flexibility under realistic threat scenarios, the proposed framework was tested against argumentative and cyber–physical attacks.

FGSM-based EEG Perturbation:

The Fast Gradient Sign Method (FGSM) was applied to EEG inputs to simulate adversarial manipulation. Results indicated a moderate accuracy drop (~3–5%), demonstrating partial robustness of the hybrid architecture [25].

Replay Attacks:

Simulated replay of previously captured network packets resulted in detectable anomalies, with the model maintaining detection accuracy above 95%.

Spoofed Apnea Events:

Artificial injection of apnea-like traffic patterns was tested. The hybrid model successfully distinguished physiological events from malicious injections due to cross-domain validation.

These results highlight the importance of integrated physiological–network modeling for improving resilience and reducing false negative risks.

### 3.8. Dataset Limitations and Generalization

The estimation was conducted on Sleep-EDF and CICIoMT2024 datasets, which, while widely used, have limitations in representing real-world diversity.

Sleep-EDF consists of relatively homogeneous subjects, lacking representation of elderly populations and patients with comorbidities [30].

Similarly, CICIoMT2024 contains synthetic traffic patterns that may not fully capture real-world IoMT variability.

These limitations may introduce risks of overfitting and reduced generalizability, particularly under heterogeneous clinical and environmental conditions.

## 4. Discussion

Sleep medicine and Internet of Medical Things (IoMT) infrastructures constitute an expeditious shift that demonstrates a fundamental paradigm shift, in the sense that it is a shift from laboratory-oriented diagnostics to continuous and home-based digital health ecosystems. But with this transformation, a two-fold challenge emerges—that of maintaining physiological fidelity in the classification of sleep stages and that of having cyber security resilience in distributed healthcare networks. We look back at this nexus and indicate the critical requirement to consider hybrid and combined AI architecture capable of potentially competing simultaneously with the two spheres in the fundamental sense.

### 4.1. Fusion Between Sleep Medicine and IoMT Ecosystems

Laboratory-based diagnostics have been overtaken as the paradigm of sleep-based diagnostics by constant home-based digital health ecologies that are integrated with the infrastructures of the IoMT. Despite this transformation enhancing accessibility and enabling long-term observing, a second problem is added: maintaining physiological precision in the classification of sleep stages while providing cybersecurity resilience to distributed healthcare networks. Sleep Health IoTs (S-HIoTs) are available as coupled cyber–physical systems that have a strong level of layer/sensory interconnection, statements, and analytics. Although signal processing can be accurate, the network is vulnerable. In this regard, there is a great need for the design of coherent AI hybrids that will be able to capture both physiological processes and network dynamics and design trustworthy and safe digital sleep monitoring devices.

### 4.2. Sequential Modeling Has Biological Meaning

Sleep architecture is dynamic and cyclic (70); structured transitions between the NREM (N1, N2, N3) and REM stages alternate at intervals of about 90–110 min. These transitions exhibit probabilistic temporal dependencies that are governed by homeostatic and circadian processes rather than random fluctuations. As a result, models that consider sleep epochs as independent observations cannot account for the biological continuity contained within sleep physiology. Bidirectional temporal modeling approaches (e.g., BiLSTM) follow this physiology-centric structure by providing context from sequential past and future epochs, simulating the human reasoning process in manual scoring. More importantly, hybrid architectures offer not only computational efficiency but also clear physiological interpretations considering the intrinsic chronobiological organization of sleep, which is preserved when combining CNN-based spatial feature extraction.

### 4.3. Cyber–Physical Interdependence in S-HIoTs

The most significant lesson of this review is that S-HIoT systems can be considered cyber–physical healthcare systems in which physiological measurements (EEG, ECG, SpO_2_) and communication systems cannot be disconnected. The weaknesses of one layer impact outgoing or incoming layers; e.g., signal attacks can cause sleeping-stage classification errors, a replay or spoofing attack can initiate a false alarm for apnea, and a denial-of-service attack may postpone important clinical alerts. There is a seeming contrast between classifications of classical intrusion detection systems in solidarity and itinerary surroundings, but sleep-stage classifiers almost never consider communication layer integrity to be an invaluable blind spot. By combining sleep analytics with anomaly detection, and with joint optimization in CNN-BiLSTM-RF, this separation will be reduced, thereby enhancing adversarial perturbation robustness, and this transferable technique will be useful for early PD diagnosis.

### 4.4. Functionary–Latency Trade-Off in Edge Deployment

Wearable sleep monitoring devices should have very tight restrictions in terms of computational and energy factors. Transformers and attention-based models have very high representational rates but may have high computational costs that render them impractical to deploy continuously at low-power settings. Hybrid CNN–BiLSTM–RF architectures represent a fair trade-off between accuracy, latency, and resource efficiency; all model variants achieved competitive performance while keeping low inference latencies suited for real-time apnea detection. Therefore, the model choice in S-HIoT systems must constitute a multi-dimensional optimization issue, which involves accuracy, false alarm latency, energy use, and memory footprint. Ultimately, this framing at the system level is fundamental to the understanding of how AI-based sleep monitoring solutions can be integrated into real-world practice on a scale basis.

### 4.5. False Alarm Reduction and Clinical Reliability

High false positive rates erode clinician trust, contribute to alert fatigue, and lower user adherence to long-term wearable monitoring devices. Stability is enhanced by the integration of multiple predictive pathways using ensemble-based decision mechanisms such as Random Forest integration with temporal persistence analysis. Hybrid approaches like these can improve the detection of sleep apnea events, estimate sleep efficiency, and enable long-term health status monitoring, thus improving the clinical benefits from S-HIoT solutions. As such, algorithmic robustness is not only a computational objective but also a factor that influences patient safety and the efficiency of a healthcare system.

### 4.6. Privacy, Ethics, and Data Governance

Sleep data encapsulates very sensitive neural and cardiac information, which is prone to unauthorized access, the misuse of data, risks associated with re-identifying individuals within archives or batches of participants in studies, and prolonged storage. We also highlight emerging privacy-preserving mechanisms such as federated learning, differential privacy, and blockchain-based auditing that enables decentralized trust management at the cost of additional computational complexity. As we look forward to developing future S-HIoT systems, a careful balance should be found between model performance and privacy preservation that complies with regulations, thus providing resource-feasible strategies that satisfies ethical-by-design approaches towards digital sleep ecosystems.

Federated learning enables decentralized model training without sharing raw patient data, preserving privacy.

Differential privacy techniques can further protect sensitive biomedical signals by introducing controlled noise during training.

### 4.7. Explainability and Clinical Interpretability

To enhance interpretability, SHAP (SHapley Additive exPlanations) is incorporated to identify feature contributions in both physiological and network domains.

Temporal attention patterns from BiLSTM further provide insight into sleep-stage transitions.

These mechanisms improve transparency, clinical trust, and regulatory compliance in medical AI systems.

### 4.8. Limitations, Failure Scenarios, and Future Directions

Despite its strong performance, the proposed framework has several limitations, listed as follows:

- Noise Sensitivity: High levels of EEG noise or motion artifacts may degrade classification accuracy.
- Missing Data: Loss of signals (e.g., SpO_2_ or ECG dropout) can affect model reliability.
- Cross-Population Generalization: Model performance may vary across different demographic groups due to physiological variability.
- Adversarial Vulnerability: Carefully crafted perturbations may affect both signal classification and anomaly detection.

These limitations highlight the need for robust preprocessing, domain adaptation, and explainable AI integration. Despite the progress made recently, several limitations remain, such as the lack of dataset diversity; limited evaluations of adversarial robustness; heterogeneous devices hindering generalization across environments, with disparate sources or properties/tasks that could exploit temporal correlations within those streams being coupled in unrecognized task spaces; explainability issues for learned representations rather than raw signals and failure when it comes to querying energy usage along different axes, thereby framing problems for not only classification but also prediction, which has various ramifications, especially in wearables, etc. Solutions to these problems will require cross-disciplinary teamwork among sleep scientists, biomedical engineers, cybersecurity experts, and AI creators. Explainable AI techniques, federated secure learning architectures, adversarial robustness testing, energy-aware model compression, and multi-modal data fusion are key future research avenues for the development of resilient, intelligent, and privacy-preserving sleep health infrastructures that can be deployed in real-world clinical settings.

## 5. Conclusions

The fast developments in wearable sensing technologies and Internet of Medical Things (IoMT) infrastructures are changing the landscape of sleep medicine, involving distributed, continuous, and home-based digital ecosystems rather than laboratory-based diagnostics. Nonetheless, the transition presents the risk of sleep health systems becoming vulnerable to cyber–physical attacks that may jeopardize physiological meaning as well as patient safety. This new intersection was critiqued in the current review, and we demonstrated that a critical gap in the existing literature is that sleep-stage analytics is not tied to network safety mechanisms.

This paper synthesizes recent advances in the areas of AI-enabled sleep classification and IoMT intrusion detection to highlight this need for integrated modeling frameworks able to holistically address physiological fidelity and cyber resilience. We presented the hybrid CNN–BiLSTM–RF paradigm framework not as just another computational structure but as a biological basis for statistical intelligence machine adaptable to secure Sleep Health IoT (S-HIoT) systems. Spatial–spectral feature learning is in line with the characteristics of EEG frequency bands, bidirectional temporal modeling mimics the repetitive and transitive nature of sleep architecture, and ensemble-based diagonal decision strategies improve robustness under non-stationary and adversarial conditions.

Through comparative analysis, we demonstrate that hybrid integration achieves competitive performance in sleep stage classification and anomaly detection compared to classic speech-processing techniques, while enabling low-complexity computation suitable for edge deployment. More significantly, the simultaneous optimization of physiology and network behavior mitigates systemic blind spots that are inherent in isolated modeling approaches. Such cross-domain validation is necessary in safety-critical healthcare contexts to mitigate the risk of misclassification from tampered signals and latency for clinical alerts from compromise in communication layers.

This work contributes to more than numerical performance improvements; the larger contribution of this work is in reconceptualizing secure sleep monitoring as a cyber–physical system problem that requires cross-disciplinary collaboration between sleep researchers, biomedical engineers, cybersecurity experts, and AI scientists. Secure digital sleep ecosystems of the future will need to adopt principles of explainable AI, privacy-preserving federated learning, adversarial robustness evaluation, and energy-aware optimization for scalable deployment whilst retaining ethical compliance and clinical trustworthiness.

Therefore, merging sleep analytics with cybersecurity is not just a technical add-on but rather a structural requirement of next-generation digital sleep healthcare. This review reconciles chronobiological modeling and network resilience under a single framework, serving as a conceptual roadmap for intelligent, secure, durable Sleep Health IoT infrastructures amenable to clinical translation.

## Figures and Tables

**Table 1:** Recent papers on healthcare monitoring systems.

Year	Paper (Short)	Verified Venue	Core Idea/Method	Dataset(s) Used	Key Results/Notes
2022	Deep Learning in IoT Intrusion Detection	Journal of Network and Systems Management (Springer Nature)	Comprehensive DL IDS review (CNN/LSTM/autoencoders, etc.), gaps and challenges	Survey/review	Strong baseline taxonomy + open problems for IoT/IoMT IDS design.
2023	Adaptive IDS in IoMT using Fuzzy-Based Learning (FST-LSTM)	Sensors (MDPI)	Fuzzy self-tuning + LSTM for IoMT traffic detection	EHMS (e-Health Monitoring System testbed) data described in the paper	Emphasizes IoMT-specific data characteristics + focuses on reducing false positives in healthcare context.
2024	Healthcare 5.0 secure system using Federated Learning + IDS + Blockchain	PeerJ Computer Science	Privacy-preserving learning (FL) + blockchain trust + IDS pipeline	(Paper-specific; described in PeerJ article)	Strong for privacy/compliance (healthcare data) and decentralized trust models.
2024	SA-FLIDS (Secure & Authenticated FL-based IDS) for Fog-IoT Smart Healthcare	PeerJ Computer Science	Federated IDS with authentication/security hardening (fog/edge healthcare)	(Paper-specific; described in PeerJ article)	Focused on fog/edge smart healthcare and security of collaborative training.
2024	Guarding Digital Health: Deep Learning for Medical IoT security	Procedia Computer Science (Elsevier/ScienceDirect)	Compares DL models (CNN, Autoencoder, Transformer, LSTM) for detection	(Not clearly shown in the ScienceDirect snippet)	Reports LSTM performance with accuracy around 97%(plus precision/recall/F1).
2025	L2D2: LSTM multi-class detection for IoMT	IEEE Access (IEEE)	Enhanced LSTM for multi-class detection/classification	CICIoMT2024	Reports ~98% accuracy for 19 classes on CICIoMT2024.
2025	HIDS-RPL: Hybrid CNN + LSTM for IoMT networks using RPL	IEEE Access (IEEE) (indexed via scholarly aggregators)	Hybrid CNN feature extraction + LSTM temporal modeling in IoMT routing settings	(Paper-specific)	Shows a common trend: CNN/LSTM hybridization for better feature + sequence learning in IoMT.
2025	Hybrid IoMT anomaly detection using GNN + Transformer	Sensors (MDPI)	Hybrid GNN/GCN + Transformer for structural + sequence dependencies	Discusses CICIoMT2024 as an IoMT benchmark and compares with ML baselines	Explicit hybrid configuration + comparative evaluation vs. LR/AdaBoost/RF.
2024	Meta-learning for ensemble IDS in IoMT	PubMed-indexed article	Meta-learning to improve ensemble IDS performance	(Paper-specific)	Motivates adaptive/transferable models for evolving IoMT threats.
2026	Adaptive hybrid IDS with lightweight optimization (IoMT)	PubMed-indexed article	Hybrid IDS + lightweight optimization; compares lightweight classifiers	CICIoMT2024	Reports high accuracy across binary/multi-class settings on CICIoMT2024.

**Table 2:** Comparative performance of representative architectures.

Model	Sleep Accuracy (%)	Network Detection Accuracy (%)	Precision (%)	Recall (%)	F1-Score (%)	Latency (ms)
CNN	94.2	92.5	93.8	92.9	93.3	55
LSTM	95.8	94.7	95.1	94.4	94.7	60
CNN + LSTM	96.9	96.2	96.5	96	96.2	52
GNN + Transformer	97.2	97.8	97.5	97.1	97.3	85
Proposed CNN–BiLSTM–RF	97.8	98.6	98.3	98.4	98.3	42

## Data Availability

The original contributions presented in this study are included in the article. Further inquiries can be directed to the corresponding author.
